# Pulsed Transcranial Red/Near-Infrared Light Therapy Using Light-Emitting Diodes Improves Cerebral Blood Flow and Cognitive Function in Veterans with Chronic Traumatic Brain Injury: A Case Series

**DOI:** 10.1089/photob.2018.4489

**Published:** 2019-02-08

**Authors:** S. Gregory Hipskind, Fred L. Grover, T. Richard Fort, Dennis Helffenstein, Thomas J. Burke, Shane A. Quint, Garrett Bussiere, Michael Stone, Timothy Hurtado

**Affiliations:** ^1^ Brain Injury Consulting, LLC, Department of Brain Research, Addison, Texas.; ^2^ InLight Medical, Medical Advisory Department, Addison, Texas.; ^3^ Revolutionary MD, Department of Medical Research, Denver, Colorado.; ^4^ CereScan Corporation, Department of Imaging Research, Littleton, Colorado.; ^5^ Colorado Neuropsychological Associates, Testing Department, Englewood, Colorado.; ^6^ University of Colorado School of Medicine, Department of Physiology (Retired), Aurora, Colorado.; ^7^ Veterans Administration Hospital, Department of Radiology, Las Vegas, Nevada.; ^8^ Penrose-St. Francis Health Services, Emergency Department, Colorado Springs, Colorado.

**Keywords:** photobiomodulation, transcranial, traumatic brain injury, quantitative, SPECT imaging, LED, cognitive function

## Abstract

***Objective:*** This study explored the outcome of applying red/near-infrared light therapy using light-emitting diodes (LEDs) pulsed with three different frequencies transcranially to treat traumatic brain injury (TBI) in Veterans.

***Background:*** Photobiomodulation therapy (PBMT) using LEDs has been shown to have positive effects on TBI in humans and animal models.

***Materials and methods:*** Twelve symptomatic military Veterans diagnosed with chronic TBI >18 months post-trauma received pulsed transcranial PBMT (tPBMT) using two neoprene therapy pads containing 220 infrared and 180 red LEDs, generating a power output of 3.3 W and an average power density of 6.4 mW/cm^2^ for 20 min, thrice per week over 6 weeks. Outcome measures included standardized neuropsychological test scores and qualitative and quantitative single photon emission computed tomography (SPECT) measures of regional cerebral blood flow (rCBF).

***Results:*** Pulsed tPBMT significantly improved neuropsychological scores in 6 of 15 subscales (40.0%; *p* < 0.05; two tailed). SPECT analysis showed increase in rCBF in 8 of 12 (66.7%) study participants. Quantitative SPECT analysis revealed a significant increase in rCBF in this subgroup of study participants and a significant difference between pre-treatment and post-treatment gamma ray counts per cubic centimeter [*t* = 3.77, *df* = 7, *p* = 0.007, 95% confidence interval (95,543.21–21,931.82)]. This is the first study to report quantitative SPECT analysis of rCBF in regions of interest following pulsed tPBMT with LEDs in TBI.

***Conclusions:*** Pulsed tPBMT using LEDs shows promise in improving cognitive function and rCBF several years after TBI. Larger, controlled studies are indicated.

## Introduction

The incidence of traumatic brain injury (TBI) has grown over the past decade. The Centers for Disease Control and Prevention estimated that >2.5 million TBI cases were reported in emergency departments in 2010, versus 1.5 million in 2001.^1^ Among returning soldiers, TBIs have been recognized as the “signature wound” of the Iraq and Afghanistan wars. Rates of depression, anxiety, and psychological symptoms are markedly elevated in TBI survivors.^2,3^ The U.S. Veterans Administration has estimated the suicide rate among Veterans is 22 per day.^4^ In addition, there has been increased media attention to the effects of TBI in various sports at all levels.

Public awareness of the devastating impact of TBI has grown, drawing the attention of clinicians and researchers. Current treatment for TBI is focused on pharmacotherapy and cognitive rehabilitation therapy. The residual cognitive and psychological impairments from TBI show minimal response to these interventions. Conversely, photobiomodulation therapy (PBMT) with light-emitting diodes (LEDs) has demonstrated positive effects on TBI in humans and animal models.^5–8^ In addition, near-infrared (NIR) light with LEDs has been demonstrated to penetrate the human skull in multiple studies.^9–11^ Hamblin has reported on the safety, convenience, and efficacy of using LEDs as a legitimate source of PBMT for several brain disorders.^7,8,12,13^

Oron^5^ demonstrated reduction in brain lesion size and improved neurological performance after a single 808 nm NIR laser treatment in a mouse TBI model. Wu et al.^6^ demonstrated improved efficacy of PBMT in mice in the 665 and 810 nm range relating to the absorption of light by cytochrome C oxidase. Xuan et al.^14,15^ demonstrated that transcranial PBMT (tPBMT) improved neurological performance and levels of brain-derived neurotrophic factor (BDNF) in mice with TBI. Quirk et al.^16^ improved the biochemical and behavioral aspects of rats using a controlled cortical impact model of TBI using transcranial LEDs at 670 nm. Ando et al.^17^ demonstrated that pulsed light at 10 Hz was superior to 100 Hz or continuous light in producing behavioral and chemical improvements in rats undergoing controlled TBI.

Naeser et al.^7^ reported two cases of TBI in humans treated with a combination of red/NIR LEDs applied bilaterally to the forehead and midline sagittal areas using LED cluster heads (total power 500 mW; 22.2mW/cm^2^ power density; 13.3 J/cm^2^). Both patients demonstrated clinical improvement, and statistical improvement in neuropsychological testing following treatment. In an open protocol case series of 11 patients with heterogeneous TBI, Naeser et al.^8^ applied similar red/NIR LED clusters for 20 min thrice per week for 6 weeks. Pre-standardized/post-standardized neuropsychological testing revealed a statistically positive trend for executive functioning, inhibition switching, and long delay free recall. Improvements in sleep and symptoms of post-traumatic stress disorder were noted. In a single patient case report using a high power (10–15 W) in-office laser pulsed at 810 and 980 nm, applied to the TBI patient's forehead in 20 sessions over a 2-month period, Henderson and Morries^18^ reported improvement in symptoms of cognition, anxiety, and depression along with qualitative improvements in regional cerebral blood flow (rCBF), as measured by single photon emission computed tomography (SPECT). Saltmarche et al.^19^ utilized 810 nm NIR pulsed at 10 Hz in a controlled, single-blind pilot study with positive effects in a population of dementia patients. In a review of the literature comparing continuous wave to pulsed wave light therapy, Hashmi et al.^20^ concludes, “this review indicates that overall pulsed light may be superior to CW light with everything else being equal.”

Based on this body of evidence, the authors determined that exposing subjects to different frequencies during each treatment using a commercially available Food and Drug Administration (FDA)-cleared device would offer a novel treatment option and add noteworthy new findings to the field.^13,17,20^ Therefore, three distinct pulse rates were used sequentially as a treatment modality.

The purpose of this study was to explore the outcome of using Pulsed Transcranial Red/NIR (PBMT) with LEDs on the neuropsychological functions of a group of 12 Veterans chronically suffering from TBI. In addition, since TBI has also been associated with decreased rCBF in numerous studies,^21–24^ this study measured possible changes in rCBF, as measured by quantitative SPECT, which has been associated with potentially positive clinical responses.

## Materials and Methods

### Design

This was an observational cohort study using a case series design that investigated the possibility that the pulsed transcranial application of red and NIR light could impact neuropsychological function and cerebral blood flow in Veterans with chronic TBI. A repeated-measures within-subjects design was used, a typical research design when investigating a new intervention.^25^ No conclusions were made regarding generalizability or cause and effect. Trial registration at clinicaltrials.gov occurred after trial completion.

### Study participants

Thirteen military Veterans diagnosed with chronic TBI were enrolled in this Western Institutional Review Board-approved study (Olympia, WA).

All participants were male Caucasians, 21–55 years [mean (*M*) = 41.25; standard deviation (*SD*) = 7.29]; years of education ranged from 12 to 18 years (*M* = 15.2 years; *SD* = 1.99). Seven were employed full time, one was self-employed, one was unemployed, one was disabled, and two did not respond. One participant dropped out of the study following treatment, resulting in a final sample of 12 Veterans (Table 1).

**Table 1.** Demographics for 12 Military Veterans with Chronic, Mild-to-Moderate Traumatic Brain Injuries Treated with Red/Near-Infrared Light-Emitting Diodes Applied Transcranially

*Patient no.*	*Age at entry*	*Years of education*	*Gender*	*Year(s) of TBI*	*Medical history for TBI*	*Work status*
1	36	16	Male	2003, 2004	Multiple IED blasts, LOC	Full time
2	31	16	Male	2009	Slipped on airstrip, head impact, LOC	—
3	46	18	Male	1985, 1989, 1991	Multiple (>8) concussions (football). Dove head first in pool, LOC	Full time
4	56	16	Male	2008	Mountain biking accident, prolonged LOC	Full time
5	48	16	Male	1993, 2011	Sports injury, orbital blowout. Bucked off horse into steel fence, multiple skull/facial fractures	Full time
6	36	12	Male	2000, 2007	Multiple (3–5) mild concussions (football). MVA, LOC	Full time
7	44	16	Male	1983, 1998	Multiple (>5) concussions (football, bull riding). Helicopter crash, LOC	Disabled
8	46	12	Male	2004, 2007	MVA resulting in head impact, LOC. Multiple IEDs	Unemployed
9	32	16	Male	2007	Multiple IEDs, LOC.	Full time
10	37	12	Male	1999–2009, 2004	Multiple IEDs, burn pit exposure. High-speed boating accident, LOC	Self-employed
11	43	16	Male	2001–2004, 2007	Repetitive head trauma (>10) concussions w/LOC (wrestling, football, boxing)	Full time
12	40	16	Male	—	MVA, LOC	—
Mean	41.3	15.2				
(SD)	(7.29)	(1.99)				

IED, improvised explosive device; LOC, loss of consciousness; MVA, motor vehicle accident; SD, standard deviation; TBI, traumatic brain injury.

### Methods

Inclusion criteria included a minimum time of 18 months since last reported brain injury for study participants. This criterion was used to ensure that any improvement in outcome measures could not be attributable to the brain's natural healing process, which can occur up to 18 months following TBI.^26^ Average TBI age was not calculated due to histories of multiple TBIs in nine study participants and memory loss in another participant. All Western Institutional Review Board (WIRB) requirements and Health Insurance Portability and Accountability Act standards were met. No paid incentives were offered, but participants were given the option to keep the treatment device.

### Outcome measures and assessment protocol

Two quantitative outcome measures were used to assess the influence of the pulsed transcranial red/NIR treatment. Instruments were administered before beginning treatment and again within 3 weeks of completing treatment. Neuropsychological tests were administered by an experienced, licensed clinical psychologist. Testing focused on the cognitive abilities frequently affected by TBI and included subscales of the California Verbal Learning Test II (CVLT-II),^27^ the Wechsler Adult Intelligence Scale IV (WAIS-IV),^28^ the trail making test B (TMT-B),^29^ and the digit vigilance test (DVT)^30^ (Appendix A). Following administration of the neuropsychological measures, each participant underwent brain SPECT scans to quantitatively measure rCBF in 138 regions of interest (ROIs). A confirmatory diagnosis from the SPECT scan was the final inclusion criterion for study enrollment.

Brain SPECT imaging was performed at an American College of Radiology (ACR)-certified CereScan facility in accordance with the 2014 ACR *Practice Guidelines and Technical Standards*. Patients were placed in a reclining chair and an IV line was established. They were allowed to acclimate in a quiet, semidarkened room with eyes open and sound-dampening headphones for 15 min. Next, the Tc99-m-labeled hexamethylpropyleneamine oxime (HMPAO) tracer was injected through the IV line and saline flushed. Scans were acquired following a 60-min washout period with a Siemens Symbia E SPECT gamma camera with low-energy high-resolution parallel-hole collimators. Counts were collected in a 64 × 64 × 64 matrix with 32 stops of 5.625 degrees each, with a zoom of 1.78. Total counts exceeded 5 million. Post-acquisition processing was done with Segami Corporation's Oasis software (www.segamicorp.com), with a Butterworth filter of 0.6 and order of 6. Chang attenuation correction was performed along with motion correction, as needed, using Cedars-Sinai MoCo software (www.cedars-sinai.edu/Patients/Programs.../Motion-Correction-MoCo.aspx). There was no post-filtering.

Qualitative SPECT data were generated using Segami's Oasis software in horizontal, sagittal, and frontal views in 4-mm sections. Images were compared to an age-matched normative database containing 59 individuals. Scans were interpreted blindly and independently to confirm the TBI diagnosis by a board-certified, nuclear qualified radiologist with extensive experience with positron emission tomography/computed tomography and SPECT interpretations, and a nuclear qualified neurologist with over 4000 successful SPECT interpretations. Concordance was 100% between the two reading physicians regarding the presence or absence of TBI. Heterogeneity of the TBI SPECT findings within the study cohort was confirmed.

Quantitative SPECT data were generated from the Oasis software and then analyzed using MIM Software MIM-neuro module, version 6.4 (www.mimsoftware.com).

### Device(s) and treatment

**FIG. 1.**
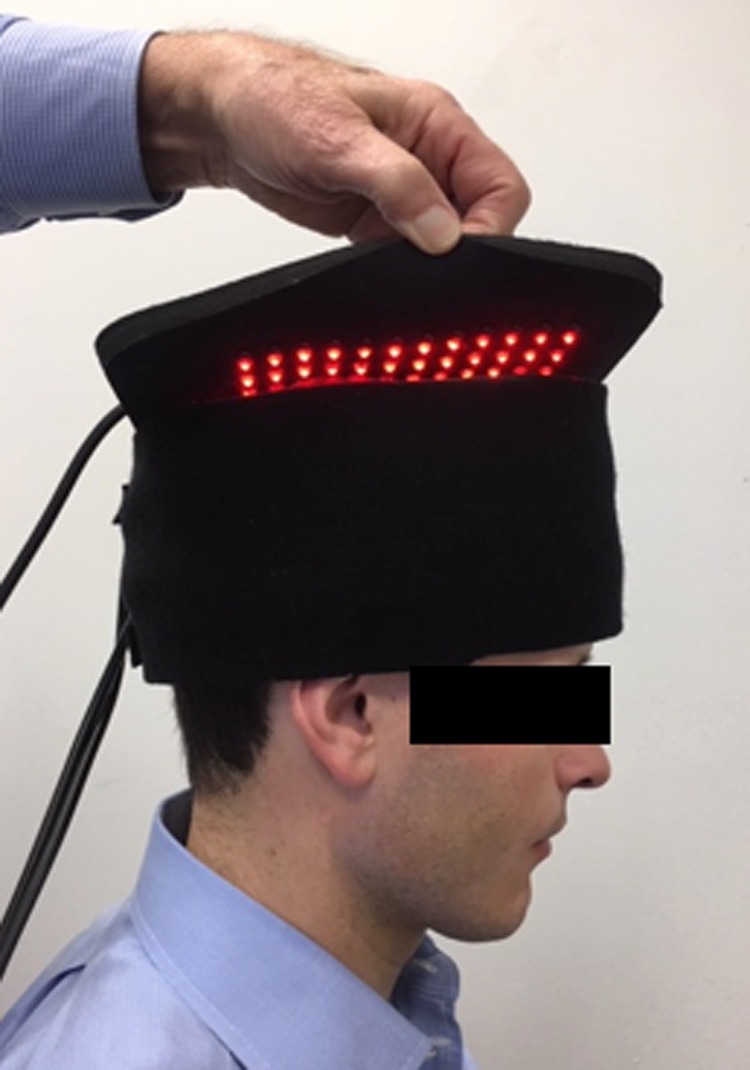
Sequentially pulsed red/near-infrared device.

Study participants were treated with an FDA-cleared red/NIR device manufactured by InLight Wellness Systems (www.inlightmedical.com). Because of the heterogeneous pattern of TBI noted in the literature and seen in the brain SPECT scans of study participants, the treatment device was designed specifically for this study to provide coverage for the entire cranium.^31^ It contained 2 separate neoprene pads embedded with alternating rows of 180 red (629 nm) and 222 NIR (850 nm) LEDs generating 3.3 W pulsed power output. One pad circled the skull and the other pad covered the top of the head, providing a total coverage area of 519 cm^2^ with an average power density of 6.4 mW/cm^2^, an average energy density of 7.7 J/cm^2^, and a peak power density of 18.3 mW/cm^2^ (Fig. 1). Published scientific evidence suggests that pulsed light is superior to continuous light application; therefore, red/NIR light was pulsed at three different frequencies (73, 587, and 1175 Hz) for 6.7 min, each at a 35% duty cycle (DC).^17–20^ DC is defined as pulse repetition rate (F) × pulse duration (PD), or DC = F × PD. Twenty-minute treatment sessions were administered thrice per week for six consecutive weeks, totaling 18 sessions at 1 of 2 medical clinics.

Participants were taken to an examination room where treatment devices were applied to the head by an experienced light therapy practitioner. The amount, color, and density of hair were not measured. Most participants had generally shorter hair. Figure 1 demonstrates the treatment device with the top pad slightly opened to view diodes. However, for treatment, the top pad was velcroed shut. Participants were interviewed regarding any concerns, side effects, and/or adverse events from prior treatments before beginning each session. Responses were recorded in a logbook at each site. No adverse effects were noted at either site. Resources were unavailable to explore sleep quality or depression issues (Table 2).

**Table 2.** InLight Wellness Systems Transcranial Photobiomodulation Therapy Device Parameters

*Source*	*LED*
Total surface area, cm^2^	519
Total red LEDs	180
Total infrared LEDs	222
Peak wavelength, red LEDs, nm	629
Peak wavelength, infrared LEDs, nm	850
Pulsed power output, W	3.3
Pulsed power density, mW/cm^2^	6.4
Pulsed energy density, J/cm^2^	7.7
Pulsed frequency first 6.7 min of 20-min session, Hz	73
Pulsed frequency second 6.7 min of 20-min session, Hz	587
Pulsed frequency third 6.7 min of 20-min session, Hz	1175
Pulse duty cycle, percentage	35
Treatment session duration, seconds	1200
Energy delivered per treatment, Joules	3994
Frequency of treatment/week	3

Light was sequentially pulsed at the three frequencies listed above, theoretically decreasing the risk of tolerance development. Further, given the heterogeneous nature of TBI, a device was designed to cover the entire cranium.^31^ Although the pulsed fluence was somewhat reduced relative to other studies at 7.7 J/cm^2^, the total Joules delivered per treatment of 3994 was 20% over that reported in a previous study due to the device's larger surface area.^8^

LED, light-emitting diode; TBI, traumatic brain injury.

## Results

### Neuropsychological testing

Paired *t*-tests were used to compare pre-treatment and post-treatment raw scores on each of 15 neuropsychological scales (*n* = 12). Results indicated a significant increase in scores on 6 of 15 scales (*p* < 0.05, two tailed). Veterans' *t*-scores significantly improved for 3 of 5 subtests of the CVLT-II (trial 5 *t*_11_ = 2.46, *p* = 0.032; short delay *t*_11_ = 2.26, *p* = 0.045; and long delay *t*_11_ = 2.35, *p* = 0.039) and 3 of 7 WAIS-IV subtests (symbol search *t*_11_ = 2.367, *p* = 0.037; coding *t*_11_ = 2.68, *p* = 0.021; and processing speed *t*_11_ = 3.188, *p* = 0.009). Nonsignificant estimates were found for the TMT-B, DVT, and remaining CVLT-II and WAIS-IV subtests (Table 3).

**Table 3.** Neuropsychological Test Results

*Test*	*Mean* t*-score pre-treatment*	*Mean* t*-score post-treatment*	t	df	p
California Verbal Learning Test II					
Trial 1	46.3	45.3	0.28	11	0.782
Trial 5	50.8	54.5	2.46	11	0.032^*^
Trials 1–5	48.6	51.3	1.77	11	0.105
Short delay	52.6	57.1	2.26	11	0.045^*^
Long delay	51.4	55.2	2.35	11	0.039^*^
Wechsler Adult Intelligence Scale IV					
Digit span	51.8	55.4	1.269	11	0.231
Letter-number sequence	49.9	54.9	2.052	11	0.065
Symbol search	48.4	53.9	2.367	11	0.037^*^
Coding	45.6	50.9	2.68	11	0.021^*^
Arithmetic	48.7	49	0.272	11	0.791
Working memory	50.1	52.3	1.178	11	0.264
Processing speed	46.8	52.7	3.188	11	0.009^*^
Trail Making Test B	42.9	47.1	0.968	11	0.354
Digit vigilance					
Speed	45.9	48.5	0.773	11	0.456
Accuracy	42.3	47.1	1.137	11	0.280

Working Memory Index derived from Arithmetic and Digit Span subtests. Processing Speed Index derived from Symbol Search and Coding subtests. *n* = 12 for all tests.

^*^ Significant improvement in pre-raw/post-raw scores (*p* < 0.05).

### Qualitative SPECT analysis

**FIG. 2.**
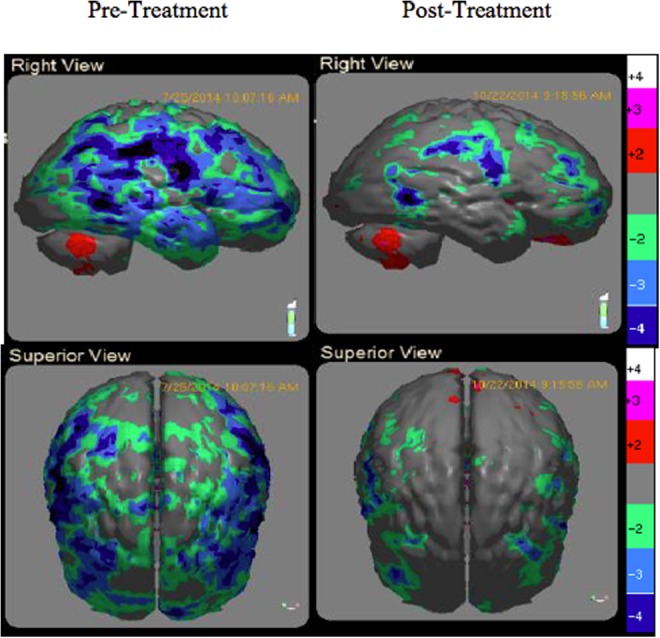
Changes in regional cerebral blood flow following 6 weeks of treatment (P3). Sagittal and superior views.

SPECT images were visually inspected to assess changes in rCBF. Each scan produced up to 75 slices and 192 images compared to a normative database by paired *t*-test to 138 different ROIs. The normative database comprised pooled data from 59 healthy individuals who served as age-matched controls in three separate, peer-reviewed scientific publications.^32–34^ Results indicated 8 of 12 (66.7%) participants showed increases in rCBF, whereas 4 of 12 (33.3%) did not. Nonetheless, these 4 showed improvements in 14/15 neuropsychological subscales. Typical SPECT images illustrating increased rCBF following treatment is displayed for one subject (patient 3) in Fig. 2.

### Quantitative SPECT analysis

Quantitative analysis of SPECT images was recently shown to provide superior results to qualitative interpretations in evaluating TBI.^35^ MIMneuro Software was used to register each image to a three-dimensional normative space before deriving counts of gamma rays per cubic centimeter (cc) of brain tissue (counts/cc) within anatomical ROIs. These counts provide a quantitative measure of rCBF (i.e., the higher the counts/cc, the greater the blood flow). Counts/cc allowed for pre-quantitative and post-quantitative comparisons of rCBF in 138 ROIs.

Identification of hypoperfused regions was assessed using resting scan pre-treatment rCBF data. MIMneuro provides *z*-scores of counts within ROIs by comparison to a normative database containing 90 individuals. A cutoff *z*-score of −1.00 was used to define an area of low rCBF (hypoperfusion). Participant resting SPECT scan results ranged from 12 to 34 ROIs with *z* ≤ 1.00. Pre-treatment and post-treatment resting scan areas of hypoperfusion were compared to evaluate the number of ROIs with increased perfusion following treatment. After treatment, rCBF increased by 47% to 92%, relative to pre-treatment low rCBF ROIs in eight participants. Four other participants showed no increase in blood flow in low rCBF ROIs at post-treatment. Among the four study participants who did not exhibit increased post-treatment rCBF, all showed increases in neuropsychological test scores (Table 4).

**Table 4.** Single Photon Emission Computed Tomography Analysis: Regions of Interest with Increased Blood Flow

*Patient no.*	*No. ROIs* z ≤ *1.00 pre-treatment*	*No. ROIs* z ≤ *1.00 post-treatment*	*No. ROIs with increased CBF post-treatment*	*% of ROIs with increased CBF post-treatment*
1	12	1	11	92%
2	24	4	20	83%
3	13	1	12	92%
4	20	3	17	85%
5	34	18	16	47%
6	15	5	10	67%
7	29	6	23	79%
8	18	18	0	0%
9	24	24	0	0%
10	28	27	1	4%
11	25	24	1	4%
12	19	6	13	68%

CBF, cerebral blood flow; ROIs, regions of interest.

Further subgroup analysis of rCBF responders (*n* = 8) demonstrated statistically increased rCBF in hypoperfused ROIs. Two-tailed paired *t*-test was used to compare pre-treatment and post-treatment gamma ray counts/cc from the non-normal ROIs. Average post-treatment counts/cc (*M* = 1,407,877.81; *SD* = 126,810.14) were greater than average pre-treatment counts/cc (*M* = 1,349,140.30; *SD* = 121,981.94). A strong positive correlation was indicated between the two variables of interest pre-treatments and post-treatments (*r* = 0.94, *p* = 0.001). Also, results revealed a significant difference between pre-treatment and post-treatment counts/cc [*t* = 3.77, *df* = 7, *p* = 0.007, 95% CI (95,543.21–21,931.82)].

In summary, these results indicated significant increases in subgroup scores on 6 of 15 neuropsychological tests and statistical increases in rCBF in 8 of 12 participants by quantitative SPECT in hypoperfused (*z* ≤ 1.0) areas of their brains, following a total of 6 h of progressively multi-pulsed tPBMT over 6 weeks. All 12 participants achieved improvement in 14/15 neuropsychological parameters; 8 of these 12 demonstrated improvement in rCBF.

## Discussion

This small case series study assesses the outcome of tPBMT with pulsed LEDs using multiple frequencies to treat Veterans with chronic TBI. Because of its capacity to noninvasively penetrate the human skull, LED-NIR light has been safely used since the late 1980s.^9–11^ tPBMT is associated with clinical improvement in subjects with neurodegenerative disease,^36^ depression,^8,37^ and TBI.^7,8,18^ Our study results support these findings.

The postulated mechanism of action of PBMT is the release of nitric oxide (NO) from the chromophore hemoglobin in red blood cells,^38^ NO synthase lining the capillary endothelial cells,^39^ and cytochrome C oxidase chromophores within mitochondria increasing adenosine triphosphate production.^40,41^ NO both activates guanylate cyclase to trigger vasodilatation and stimulates angiogenesis through the process of photobiomodulation.^42^ PBMT studies also suggest that NO increases reactive oxygen species, anti-inflammatory molecules, and neuroprotective factors (e.g., BDNF).^14,15,43^

Increased rCBF was visualized with qualitative SPECT and measured with quantitative SPECT 1–4 weeks following the cessation of tPBMT therapy. These findings suggest a positive effect on both cellular neuropathophysiological processes and the production of ongoing neuroprotective factors, possibly including BDNF.^14,15^

Six of 15 neuropsychological tests showed significant improvements in memory, concentration, and cognitive processing speed and all, but one test, showed positive outcomes. All study participants verbally reported substantial reductions in many of their TBI symptoms.

Finally, SPECT data clearly showed rCBF improvements in 8 of 12 participants. It is unclear why the other four participants showed no increase in rCBF following treatment, despite positive clinical responses. Since 3 of the 4 (75%) who did not increase rCBF had a higher incidence of blast injuries, it is possible that different light dosing parameters exist for those who sustain blast versus acceleration/deceleration injuries. Explosive blast TBI (bTBI) shares many clinical presentations with closed-head TBI. However, bTBI has such unique features as early cerebral edema, prolonged cerebral vasospasm, and different diffuse axonal injury that support the concept that bTBI is a separate and distinct type of TBI.^44^ These findings should be considered in designing future clinical trials.

### Future studies

These preliminary results strongly support future studies involving larger numbers of subjects using sham and control groups with randomization and double-blinding procedures. Also needed are studies differentiating between bTBI and acceleration/deceleration TBI responses to light therapy, and longitudinal measures at 6 and 12 months post-treatment without interval intervention to assess the long-term efficacy of tPBMT. Finally, studies comparing focal versus global applications of tPBMT at varying doses and various pulsed frequencies should be explored.

### Study limitations

The limitations of this study include the use of a small, voluntary sample with no control or sham treatment groups for comparison. This was a case series design in which all participants received the same treatment and blinding of participants and clinicians administering the treatment was not performed. Unconscious experimenter bias and placebo effects must be controlled in future studies. Alternate forms of neuropsychological assessments were not used due to budget, personnel, and time constraints, introducing potential practice effects. A third limitation is the possibility that regression to the mean could partially explain the results of the quantitative SPECT analyses due to the selection criteria cutoff of *z* ≤ 1.00. Theoretically, removal of selection criteria would alleviate this issue; however, it is one of the best practice identifiers for the decreased rCBF associated with TBI.^21–24,45^ Another possible limitation is the reproducibility of SPECT, although research shows SPECT reproducibility to be between ±1.3% and 5%.^46^

## Conclusions and Summary

These study results corroborate previous research using tPBMT with LEDs for treatment of chronic TBI.^7,8,13^ This was the first study of its kind to utilize sequentially pulsed LED technology, as well as quantitative SPECT data to measure rCBF responses to tPBMT with red/NIR LEDs administered through an affordable FDA-cleared device, as suggested in other studies.^13^ As innovative treatments for neuropsychiatric dysfunctions are developed, quantitative brain biomarkers should be utilized to augment the evaluation of scientific and medical efficacy.

It appears the use of tPBMT with pulsed LEDs may improve cognitive function and decrease the rCBF deficits associated with chronic TBI. The statistical improvements in so many aspects of neuropsychological functioning and brain perfusion with a sample of 12 suggest a treatment effect that is worthy of further investigation. Considering the cost/benefit ratio and convenience of LEDs, the economic, health, and social impact of tPBMT with LEDs in the treatment of TBI could be substantial.

## Appendix

**Appendix A.** Neuropsychological Test Descriptions

*Test*	*Description*	*Cognitive abilities*
California Verbal Learning Test II	Verbal list-learning task that involves learning of 16 items presented over 5 trials.	Auditory learning; short-term memory; long-term memory
Wechsler Adult Intelligence Scale IV	*Digit Span:* Test of auditory attention and span of immediate verbal recall in which number lists are presented in series of three digits, then four digits, and so on to a series of nine digits.	Working memory; auditory attention
	*Coding:* Test of complex visual attention, visual-motor speed and coordination, and visual scanning.	Cognitive processing speed; perceptual reasoning
	*Letter-Number Sequencing:* Task that requires keeping items in memory long enough to rearrange their order.	Working memory; auditory attention; sequencing
	*Arithmetic:* Test of arithmetic problems presented in story form according to level of difficulty.	Working memory; auditory attention; concentration
	*Symbol search:* Test of symbol search and identification.	Visual attention; visual scanning; concentration
Trail Making Test (Part B)	Test of visual scanning, visual-motor tracking, alternating attention, and logical sequencing.	Visual motor; visual spatial; mental flexibility
Digit Vigilance Test	Test of visual attention, visual scanning speed, psychomotor speed, and visual scanning accuracy.	Executive function, vigilance, and alertness
